# A 40-bp VNTR polymorphism in the 3′-untranslated region of *DAT1/SLC6A3* is associated with ADHD but not with alcoholism

**DOI:** 10.1186/s12993-015-0066-8

**Published:** 2015-06-11

**Authors:** Omar Šerý, Ivo Paclt, Ivana Drtílková, Pavel Theiner, Marta Kopečková, Petr Zvolský, Vladimir J. Balcar

**Affiliations:** Laboratory of Neurobiology and Molecular Psychiatry, Department of Biochemistry, Faculty of Science, Masaryk University, Kotlářská 2, 611 37 Brno, Czech Republic; Institute of Animal Physiology and Genetics, Academy of Science, Veveří 97, 602 00 Brno, Czech Republic; Department of Psychiatry, 1st Faculty of Medicine, Charles University, Ke Karlovu 11, 121 08 Prague, Czech Republic; Department of Psychiatry, Masaryk University, Faculty of Medicine, Kamenice 5, 625 00 Brno, Czech Republic; Laboratory of Neurochemistry, School of Medical Sciences (Discipline of Anatomy and Histology) and Bosch Institute, Sydney Medical School, The University of Sydney, Sydney, NSW 2006 Australia

**Keywords:** Hyperkinetic disorder, Polymorphism, Alcoholism, Dopamine transporter, miRNA

## Abstract

**Background:**

ADHD and alcoholism are psychiatric diseases with pathophysiology related to dopamine system. DAT1 belongs to the SLC6 family of transporters and is involved in the regulation of extracellular dopamine levels. A 40 bp variable number tandem repeat (VNTR) polymorphism in the 3′-untranslated region of *DAT1/SLC6A3* gene was previously reported to be associated with various phenotypes involving disturbed regulation of dopaminergic neurotransmission.

**Methods:**

A total of 1312 subjects were included and genotyped for 40 bp VNTR polymorphism of *DAT1/SLC6A3* gene in this study (441 alcoholics, 400 non-alcoholic controls, 218 ADHD children and 253 non ADHD children). Using miRBase software, we have performed a computer analysis of VNTR part of *DAT1* gene for presence of miRNA binding sites.

**Results:**

We have found significant relationships between ADHD and the 40 bp VNTR polymorphisms of *DAT1/SLC6A3* gene (*P* < 0.01). The 9/9 genotype appeared to reduce the risk of ADHD about 0.4-fold (*p* < 0.04). We also noted an occurrence of rare genotypes in ADHD (frequency different from controls at *p* < 0.01). No association between alcoholism and genotype frequencies of 40 bp VNTR polymorphism of *DAT1/SLC6A3* gene has been detected.

**Conclusions:**

We have found an association between 40 bp VNTR polymorphism of *DAT1/SLC6A3* gene and ADHD in the Czech population; in a broad agreement with studies in other population samples. Furthermore, we detected rare genotypes 8/10, 7/10 and 10/11 present in ADHD boys only and identified miRNAs that should be looked at as potential novel targets in the research on ADHD.

## Background

Dopamine and dopaminergic signalling have been implicated in aetiologies of several psychiatric and neurological disorders including attention-deficit/hyperactivity disorder (ADHD) and alcoholism. Dopamine transporter DAT1 is thought to be the main regulator of synaptic and extracellular levels of dopamine and, consequently, the corresponding gene *DAT1/SLC6A3,* has been among the most often studied candidate genes in relation to ADHD [1]. This is perhaps less so in the case of alcoholism [2, 3].

DAT1 is a Na^+^ and Cl^−^ dependent transporter of the solute carrier family 6 (SLC6A3). The transport process is active, deriving its free energy from the transmembrane concentration gradients of Na^+^ and Cl^−^ that are generated by Na^+^/K^+^ATPase and potassium chloride symport [4, 5]. The major physiological role of the DAT1 is the regulation of the extracellular dopamine by means of rapid transport (reuptake) of dopamine back into the dopaminergic presynaptic terminals.

Dopamine transport is a well-established target of potent psychoactive compounds including cocaine, amphetamines and PCP derivatives suggesting that the removal of dopamine from extracellular space is a very important physiological process [5–8]. Whereas cocaine and methylphenidate are blockers of dopamine uptake, amphetamine and methamphetamine can displace dopamine as substrates of the dopamine transporter and can trigger off release of dopamine from intracellular stores (reviews: [6, 7]). Two of the most commonly used ADHD medications, Ritalin™ (methylphenidate) and Adderall™ (combination of D- and L-amphetamine formulations) target dopamine transporters [7, 8].

*DAT1/SLC6A3* gene encoding DAT1 comprises 15 exons spanning 60 kb on chromosome 5p15.32, encodes 620 amino acids with 12 putative transmembrane domains and a large second extracellular loop with several putative N-glycosylation sites that need to be glycosylated to transport dopamine (review: [5]).

The 3′-untranslated region (3′-UTR) *DAT1/SLC6A3* polymorphism is a 40 bp VNTR (variable number tandem repeat) repeated between 3–13 times with the 9 and 10-repeat allele being the most frequently found in the human population. This polymorphism has been linked to various disease phenotypes involving disturbed regulation of dopaminergic neurotransmission [9].

DAT1 is primarily expressed in dopamine neurons. DAT1 immunoreactivity is concentrated in somatodendritic and terminal fields of mesencephalic dopamine neurons with most pronounced immunoreactivity in striatum and nucleus accumbens [10, 11]. Both of these brain regions are potentially involved in the aetiology of alcoholism and ADHD. However, a subpopulation of dopaminergic neurons residing in ventral tegmental area (VTA) projects also to various limbic structures including anterior cingulate cortex (ACC) and other cortical areas such as prefrontal cortex (PFC) [12]. Both ACC and PFC are thought to play part in pathogenesis of ADHD but may also be involved in, or affected by, alcoholism.

In our previous studies we found associations between ADHD, *ANKK1* (*DRD2*) gene polymorphism and other candidate genes [13–15] and an association between the alcoholism and *COMT* gene [16]. In our study of alcoholic patients, we found protective effect of the A allele of BDNF Val66Met polymorphism (rs6265) on colour vision deficiency induced by long-term excessive alcohol intake and probably related to NMDAR neurotoxicity [17].

In the present study we have been using well characterized groups of Czech subjects similar to those used in our previous studies [13–16]. We now report the results of the analysis of the relationship between ADHD, alcoholism and *DAT1/SLC6A3* gene polymorphism in a subset of a population sample numbering a total of 1312 persons.

## Materials and methods

### Selection of ADHD patients and controls

The total of 471 mutually unrelated persons of Czech ethnicity were included into ADHD study, 218 of them children with ADHD (11 girls and 207 boys; for diagnostic criteria *vide infra*) and 253 control subjects of corresponding age (47 girls and 208 boys). All subjects were apparent Caucasians. Children treated for ADHD (in both outpatient and inpatient settings) in the Child Ward of the Department of Psychiatry of the University Hospital in Brno, in the Children’s Psychiatric Hospital in Velká Bíteš, and in Department of Child Psychiatry, 1st Faculty of Medicine, Charles University, Prague were included in the study group. Control group were students collected as volunteers from elementary schools in Brno (Brunn) and Praha (Prague).

Children aged 7 – 13 years were included in the study (mean age ± SEM, 10.0 ± 1.7). In each case a written informed consent was obtained from the child’s legal guardian. Excluding criteria for participation in the study for either of the groups were congenital genetic disorders, epilepsy, mental retardation, schizophrenia, pervasive developmental disorders and serious medical illness.

In the group of ADHD-patients a detailed child-psychiatric clinical examination was conducted with a diagnostic assessment based on criteria of the 10th revision of International Classification of Diseases – ICD 10 [18] as well as the DSM-IV [19].

The following questionnaires were used in the selection and evaluation of the control subjects CTQ – Conners Teacher Rating Scale [20] and CPQ – Children’s Parent Questionnaire [21]. Only male participants who scored both 9 points or less in CPQ questionnaire and 7 points or less in CTQ questionnaire were included in the control group. In addition, all control subjects were examined by a qualified psychiatrist who administered a structured questionnaire based on criteria specified in DSM-IV [19]. Thus any individuals significantly affected by ADHD or any other mental disease would have been eliminated from the group.

### Evaluation of ADHD patients

We used the Shape Discrimination Test (TDT, *Test diskriminácie tvaru*; [22]) for assessment of attention. The test is based on determining the level of accuracy and speed while differentiating among various shapes in a given template. It requires constant mental focus on a monotonous task, therefore, the test is considered as suitable for evaluating attention. The test makes it possible to rate two criteria of achievement – speed (TDTr) and accuracy (TDTp). The results were corrected for age, gender and the level/type of education to date and standardized according to the test manual. Using a simple algorithm, also specified in the test manual, the probands could be assigned to one of the attention-deficit group.

Impulsivity was tested by the TE-NA-ZO test (Test of finding of familiar figures, *Test nachádzania známych obrázkov*; [23]). This test is based on a theoretical model of cognitive style, represented by two components – impulsivity (imp) and efficiency (ef). Both imp and ef are measured on a scale of 1–10 points. Scores 1–2 represent very high ef and very low imp (with a significant amount of thoughtful decision making), scores up to 4 would still be indicative of high ef and low imp but 5–6 points are in the region of ambivalence. Scores above 7 would mean low ef and high imp with 9–10 indicating the low and high extremes on both scales (efficiency and impulsivity, respectively).

In addition we used four tests from the neuropsychological battery for psychological assessment of the Neurobehavioral Evaluation System (NES2) [24]: Finger Tapping, Hand-Eye Coordination, Visual Digit Span and Switching Attention. Finger Tapping evaluates motor speed during a given time interval (30 s in this study). The test is done separately on dominant and non-dominant hand as well as on both (alternating) hands. Hand-Eye Coordination test evaluates visual-motor coordination. The specific task is to use a joystick and try to keep the cursor on the monitor pointed to a dot moving at a constant speed along a sinusoid curve. Visual Digit Span evaluates short-term memory and attention. The proband is required to recall a row of displayed numbers, in a manner similar to a subtest used in the Wechsler Intelligence Scale. Switching Attention Test is used to assess selective attention. It quantifies the reaction to changes in the direction of an arrow displayed on the monitor; both the number of misses (dir) and the reaction time (RT) are measured and included in the evaluation.

### Collection of DNA samples from ADHD patients and control subjects

Samples of buccal tissues were collected using Flocked Swabs (Copan, Italy) after the diagnosis was established. DNA from swabs was isolated by using the UltraClean Tissue DNA (MoBio, USA) commercial kit.

### Alcoholic patients and controls

Alcohol dependent subjects were recruited from General University Hospital in Prague, and several psychiatric hospitals (Jihlava, Červený Dvůr, Lojovice, and Prague). In total, 441 alcohol-dependent subjects (302 males and 139 females) were diagnosed to be alcohol-dependent (mean age ± SEM, 45.0 ± 8.7 years). The diagnosis of alcohol dependence was based on a clinical assessment according to DSM-IV criteria [19], the self-administered Michigan Alcoholism Screening Test (MAST) [25], and the CAGE questionnaire [26]. Responses to the CAGE questionnaire are scored 0 for “no” and 1 for “yes” designed so that higher scores indicate higher probability of problem drinking. A total score of 2 or greater is considered a clinically significant indicator of alcohol dependence. Additionally, all alcoholic patients were subjected to detailed questioning as to the occurrence of delirium tremens, evidence of alcohol-related problems with police, convictions for driving under the influence of alcohol, presence of social instability and tobacco smoking.

The control group comprised 400 mentally healthy subjects (150 males and 250 females) who declared themselves free of tobacco smoking, alcohol drinking and illegal drug use (mean age ± SEM, 43.3 ± 8.7 years). As an additional safeguard, all subjects were examined by a structured questionnaire based on criteria specified in DSM-IV [19]. This would have detected and eliminated any person with a previously undeclared addiction (whether to alcohol, nicotine or any other drug of abuse) as well as anybody with a significant mental illness. The control subjects were recruited from the same area as the case group (Somatic and Transfusion Departments of General University Hospital in Prague, Transfusion Department of University Hospital Brno, Czech military personnel, universities, manufacturing industry, National Theatre Brno etc.). Control group was screened for alcoholism using the same MAST and CAGE questionnaires as in the case of alcoholic patients.

### Collection of samples from alcoholic patients and controls

Samples of venous blood (containing EDTA to prevent coagulation) were collected from all of the alcoholic and control subjects. DNA was extracted from 200 μL of whole blood using the UltraClean Blood DNA Isolation Kit (Mobio, USA).

### DNA analysis

The detection of 40-bp VNTR polymorphism of *DAT1* gene was performed using a method described by Shinohara *et al.* [27]. PCR reaction contained: 5 ng of DNA, Kapa2G Fast ready Mix (Kapa Biosystems), and 100 nM primers 5′- tgt ggt gta ggg aac ggc ctg ag - 3′ and 5′- ctt cct gga ggt cac ggc tca agg - 3′. Amplification was revealed in thermal cycler Techne Touchgene Gradient. After initial denaturation for 2 min at 94 °C, DNA was amplified in free steps: denaturation for 30 s at 94 °C, annealing for 20 s at 66 °C, and extension for 20 s at 72 °C. Final extension of 5 min at 72 °C was used. For the detection of PCR fragments, gel electrophoresis on 2 % agarose gel EliPhore (ELISABETH PHARMACON, Czech Republic) stained by ethidium bromide was used. We detected 440 bp fragment (9 repeat allele), 480 bp fragment (10 repeat allele), and 520 bp fragment (11 repeat allele).

### Statistical analyses

CSS Statistica Software (StatSoft, USA) was used for most of the statistical evaluations. Contingency tables were constructed from the genotyping data. The chi-square test was used for the comparison of genotype frequencies in the groups; Fisher’s exact test was used for the allelic frequencies comparison. The statistical difference between genotype frequencies in ADHD group was calculated using Fisher’s exact test by R software [28]; this test was chosen because it could handle the absence (zero number) of atypical genotypes in the control group. Subtypes with different cognitive performance profiles were identified by Quick Cluster (k-means) method. The statistical significance of the differences among the subtypes of subjects based on their performance in TDT, TE-NA-ZO and NES2 tests was determined using Mann–Whitney *U* Test.

### In silico analysis

We have performed a guided computer analysis of *DAT1* gene by miRBase software [29]. VNTR part of *DAT1* from NCBI (Reference Sequence NM_001044.4) has been analysed.

## Results

### ADHD

Genotyping was successful in all 471 ADHD patients and controls (Table 1). In addition to the common 9/9, 9/10, and 10/10 genotypes we have also found rare genotypes 8/10 (one boy), 7/10 (two boys) and 10/11 (three boys). These rare genotypes have been found only in ADHD boys and have been seen neither in ADHD girls nor in any of the controls. Genotype distribution of 40-bp VNTR polymorphism between ADHD and controls was found to be significantly different using Fisher’s exact test (*P* < 0.01). The 9/9 genotype appeared to reduce the risk of ADHD about 0.4-fold (Odds Ratio = 0.3929; 95 % CI of OR = 0.1521 till 1.0149, Risk Ratio = 0.4096; 95 % CI of OR = 0.1644 till 1.0206, *p* < 0.04). We also detected a difference in the rare genotypes frequency between ADHD and controls (*p* < 0.02). There was no significant difference in 10/10 genotype frequency between ADHD patients and controls (*p* = 0.2).

**Table 1:** Genotype distribution of 40-bp VNTR polymorphism in ADHD and controls

Genotype	9/9	9/10	10/10	Atypical/rare genotypes	Total	p
ADHD	6	81	125	6	218	0,0079
controls	17	101	135	0	253	

The statistical difference between genotype frequencies was calculated using Fisher’s exact test

We have not found an association between 10-repeat allele and divided subgroups of ADHD subjects characterized by very high impulsivity or high inattention. We have not found any association between high-scored ADHD subjects and low-scored ADHD subjects in neuropsychological tests and the 10-repeat allele.

### Alcoholism

Genotypes were analyzed in all 841 alcoholics and controls (Table 2). Rare genotypes 8/8, 8/10 and 8/11 of VNTR polymorphism of *DAT1* gene were found in alcoholic patients and controls in similar numbers. Rare genotype 9/11 was found in male controls only. When we compared genotype frequencies between alcoholics and control subjects, we have not observed any statistically significant difference whether we analysed the group as a whole or separately as male and female groups. When we compared *DAT1* genotypes and alleles with data from personal histories of the alcoholic patients, we have not been able to find any statistically significant relationships between *DAT1/SLC6A3* polymorphism and the occurrence of delirium tremens, evidence of problems with police, convictions for the driving a motor vehicle under the influence of alcohol, social instability and tobacco smoking.

**Table 2:** Genotype distribution of 40-bp VNTR polymorphism of DAT1 in alcoholics and controls

Genotype	9/9	9/10	10/10	9/11	Atypical/rare genotypes	Total	*Χ* ^2^	p
controls	17	141	232	3	7	400	3,69	0,449
alcoholics	22	158	252	0	9	441		
Male controls	7	50	85	3	5	150	7,18	0,127
Male alcoholics	18	106	172	0	6	302		
Female controls	10	91	147	0	2	250	1,64	0,65
Female alcoholics	4	52	80	0	3	139		

### In silico analysis

Using miRBase software [29] we found in VNTR part of DAT1 gene that is transcribed as part of mRNA the following miRNA binding regions: mir-1972, miR-30b-5p, miR-1301 and miR-6070.

## Discussion

### ADHD: General considerations and possible role of changes in DAT1/SLC6A3

Patients with ADHD are known to have morphological abnormalities (mainly decreased size) in the cerebrum. Furthermore, the dorsolateral prefrontal cortex (DLPFC), anterior cingulate cortex (ACC) as well as their connections with striatal and parietal cortical regions appear to be hypoactive [30] (reviews: [31–33]. These regions and pathways are thought to be important in the mechanisms of maintaining and shifting attention as well as processing and filtering sensory input and, therefore, would seem to be critical in the aetiology of ADHD; interestingly, methylphenidate normalizes the hypoactivities observed in ADHD (reviews: [34, 35]). As the primary target of methylphenidate is dopamine transport including the dopamine transporter DAT1 [1, 6–8] changes in dopamine transport (perhaps involving altered expression of DAT1) would seem to be the most probable culprit in the development of ADHD.

It has been shown that distinct variations in *DAT1/SLC6A3* are associated with altered functions of brain regions involved in ADHD [36] and molecular genetic studies have focused on *DAT1/SLC6A3* as one of the “hot genes” in ADHD (review: [37]). Specifically, the relationship between VNTR polymorphism of *DAT1/SLC6A3* gene and ADHD, as well as the relationship between this polymorphism and alcoholism, have been previously studied (reviews: [3, 38, 39]) and, furthermore, meta-analysis of the influence of VNTR polymorphism of *DAT1/SLC6A3* gene on ADHD indicated a statistically significant relationship (OR = 1.12, 95 % CI: 1.00-1.27) [40]. Interestingly, chronic exposure of mice to alcohol has been shown to lead to epigenetic modification of dopamine transport resulting in an ADHD-like phenotype in the offspring [41]. Translated to the human condition, this could provide a link between inherited predisposition to alcoholism and ADHD.

The above considerations might appear less conclusive in the context of larger genome wide association studies (GWAS), particularly after meta-analysis [42]; see also critical comments in a more recent review [43]. Authors of the meta-analytical study [41], however, pointed out that GWAS samples may have been hurt by ethnic and cultural heterogeneity and called for additional collaborative efforts in collecting and analysing relevant ADHD genetic data. The present data have been obtained from a sample of a distinct and relatively homogenous population thought to be representative of the European Caucasians [44]; the sample may have been also less heterogeneous than those used in GWAS studies, particularly in terms of factors such as food habits or life style in general (for more detailed discussion see [42]).

### Polymorphisms in 3′-UTR of VNTR in DAT1/SLC6A3 gene and ADHD

We have detected significantly different *DAT1/SLC6A3* genotype frequencies distribution between ADHD children and controls. Allele 10 has not been shown to be a “risk” allele for ADHD, as previously indicated by meta-analysis [39]. Additionally, a meta-analytical survey of human single photon emission computed tomography studies yielded no evidence of any significant association between polymorphisms of the VNTR in *DAT1/SLC6A3* gene and individual variations in DAT1 availability in the human striatum [45]. We have, however, identified rare genotypes 8/10, 7/10 and 10/11 as “risk” - (*p* < 0.01) while the 9/9 genotype turned out to be protective. It is interesting to note that rare genotypes 8/10, 7/10 and 10/11 were identified in only six ADHD boys while in other 1306 persons (including alcoholic patients and controls) these genotypes were completely absent. The results of VanNess *et al.* [9] indicated that first 800 bp of the *DAT1* 3′UTR region was associated with a decreased *DAT1* transcription, mRNA stability and translation. The presence of 10-repeat allele resulted in the DAT1 binding site density elevated by approximately 50 % compared to the 9-repeat allele. Fuke *et al.* [46] suggested that the 10-repeat allele is related to a greater gene expression. The 10/10 genotype has been associated with increased dopamine concentration in the CSF, lower IQ, several neuropsychological and neurophysiological functions (review: [47]). An interesting view on DAT1 involvement in ADHD and other mental disorders put forward by Kanno and Ishiura [48]. They found that HESR family members had inhibitory effect on DAT gene expression depending on the number of repeats in the VNTR domain. The HESR family genes *HESR1, HESR2 and HESR3* were identified as the hairy/enhancer split-type basic helix-loop-helix genes. These genes have been shown to be direct transcriptional targets of the NOTCH signalling pathway that is linked to neurodevelopment. *HESR1* and *HESR2* inhibit *DAT1* expression depending on the VNTR allele by 25–50 %. The same authors reported that the presence of androgen receptor influenced inhibition of *DAT1* by *HESR1* and *HESR2* depending on *DAT1* VNTR polymorphism. Allele 9 decrease and 10 allele increase *DAT1* expression in the presence of androgen receptor [49]. HESR does not seem to have been recently investigated in clinical studies of ADHD.

### Non-coding RNA and possible involvement of epigenetics in the DAT1/SLC6A3 polymorphisms in ADHD

An alternative explanation for the relationship between *DAT1* VNTR polymorphism and ADHD is related to the regulation of gene expression by miRNA. Recent developments very much broadened our knowledge and understanding of the mechanisms necessary for orderly gene expression (recent review: [43]). Each cell produces large amounts of RNA and not all of this RNA serves as a template for the production of proteins. Hitherto unknown species of so called “non-coding RNAs” (ncRNA) have been discovered and some of them have been identified as miRNA. The main role of miRNA seems to be to specifically intervene in the mechanisms of gene expression [50]. These recently discovered ncRNA’s (including specific miRNA’s) are now methodologically accessible to targeted genetic studies in humans (review: [43]).

In the case of *DAT1* it has been predicted that it is targeted by 49 miRNAs identified in miRDB database [29]. Accordingly, we have performed a guided computer analysis of VNTR part of *DAT1* gene that is transcribed as part of mRNA. VNTR part of *DAT1* from NCBI (Reference Sequence NM_001044.4) has been analysed by miRBase software [29]. We have found the following miRNA binding regions in VNTR part of *DAT1* gene: mir-1972, miR-30b-5p, miR-1301 and miR-6070. Mir-1972 was cloned in acute lymphoblastic leukaemia but its role is not known [51]. MiR-1301 mentioned in the context of tumorogenesis, circulating leptin levels and pre-eclampsia [52]. MiR-6070 has been identified by Voellenkle *et al.* [53] as being expressed by endothelial cells exposed to hypoxia. MiR-30b-5p may act as a suppressor of cardiac hypertrophy [54]. These miRNAs should be looked at as novel targets of the research on ADHD because they could be involved in the regulation of *DAT1/SLC6A3* expression which would then be influenced by the VNTR polymorphism identified in the present study as associated with ADHD.

It has been shown that epigenetic mechanisms such as the cytosine methylation can affect the formation of ncRNA as well and, in turn, ncRNA affects methylation of cytosine [55]. Cytosine methylation is affected by a wide range of factors like age, gender, race, food, dietary amount of vitamins B6 and B12, by stress and toxic substances (review: [42]). Given that the aetiology of ADHD is complex, has a substantial environmental component and involves significant comorbidity [30, 56–58], the role of epigenetics in ADHD should be given much more attention in future studies. In fact, Womersley *et al.* [59] reported a relationship between unpredictable maternal separation in early postnatal life and altered dopaminergic system in spontaneously hypertensive rat i.e. in an animal model of ADHD [60]. The altered dopaminergic system was characterised by reduced presence of *DAT1/SLC6A3* on the cell surface and/or lower affinity of DAT for dopamine; given that these changes were produced by early postnatal events, it is very likely that epigenetic mechanisms, including DNA methylation, were involved (see [43] for a review).

### 3′-UTR VNTR polymorphism in DAT1/SLC6A3 gene and alcoholism

Human neuroimaging studies have identified neuroadaptive changes in the brain in response to chronic alcohol consumption. The relevant neural circuits are anchored in midbrain areas and their dopaminergic and glutamatergic projections [61, 62] to other brain structures commonly associated with addiction, such as the ventral striatum, striatal-pallido-thalamic loops, and prefrontal cortices [63]. *DAT1/SLC6A3* has been previously recognized as a candidate gene in the alcoholism pathogenesis in striatum and other brain areas related to reward system. The relationship between *DAT1/SLC6A3* and alcoholism may be complex and could involve additional interactions with other genes. We haven’t been able to find any clear association between *DAT1/SLC6A3* VNTR polymorphism and alcoholism (or any particular manifestations of it) in the population samples that we have analysed and this is in agreement with a previous meta-analytical study by van der Zwaluw [3]. It may imply that the associations identified as statistically significant in the current study are specifically related to ADHD.

## Conclusion

We have identified significant differences between juvenile ADHD patients and corresponding controls in *DAT1/SLC6A3* gene encoding DAT1 dopamine transporter. The differences were of a VNTR polymorphism type in an untranslated region of the gene. Preliminary *in silico* analysis indicated that the region is potentially rich in miRNA’s binding sites. These miRNA’s may be of importance in the regulation of DAT1 expression. The present study has detected no significant differences between alcoholic patients and controls thus suggesting that the studied variations of *DAT1/SLC6A3* (and possible resulting changes in DAT1 expression) could be more closely associated with ADHD than alcoholism.
